# Case Report: Response to ipilimumab and nivolumab in a patient with adrenocortical carcinoma

**DOI:** 10.3389/fonc.2023.1242560

**Published:** 2023-09-08

**Authors:** Rebecca Charles, Divine Madhu, Alexander Powles, Adam Boyde, Owen Hughes, Nagappan Kumar, Sing Yu Moorcraft

**Affiliations:** ^1^ Pathology Department, University Hospital of Wales, Cardiff, United Kingdom; ^2^ School of Medicine, Cardiff University, Cardiff, United Kingdom; ^3^ Radiology Department, Swansea Bay University Health Board, Swansea, United Kingdom; ^4^ Department of Urology, University Hospital of Wales, Cardiff, United Kingdom; ^5^ Cardiff Liver Unit, University Hospital of Wales, Cardiff, United Kingdom; ^6^ Oncology Department, South West Wales Cancer Centre and Swansea University Medical School, Swansea, United Kingdom

**Keywords:** adrenocortical cancer, adrenal cancer, immunotherapy, ipilimumab, nivolumab

## Abstract

**Background:**

Adrenocortical carcinoma (ACC) is a rare malignancy with limited treatment options. The evidence for the use of immunotherapy in ACC has been conflicting, with overall response rates ranging from 6 – 33%.

**Case presentation:**

We describe the case of a 32 year old patient who was initially thought to have an inoperable clear cell renal cell carcinoma and was treated with immunotherapy with ipilimumab and nivolumab. The patient had an excellent partial response to treatment. Further work-up prior to consideration of surgery demonstrated that the tumour was an ACC, rather than a renal cancer. She had a right adrenalectomy and right hepatectomy, achieving an R0 resection and remains disease-free one year after surgery.

**Conclusion:**

This case illustrates the challenge of diagnosing ACC, and that doublet immunotherapy with ipilimumab and nivolumab can have significant clinical efficacy in ACC.

## Introduction

Adrenocortical carcinoma (ACC) is a rare malignancy, with an incidence of 0.5 – 2 new cases per million people per year (1). The management of localised ACC is surgery, followed by consideration of adjuvant treatment with mitotane (2). It is important to achieve a margin-free complete resection (R0 resection) as resection status is an important prognostic factor (3).

For patients with locally advanced or metastatic disease that is not amenable to complete resection, the current treatment options are either single agent mitotane or mitotane in combination with etoposide, doxorubicin and cisplatin (2). The survival benefit from systemic treatment is limited, and treatment is often associated with significant toxicities (4, 5).

Over the last few years, immunotherapy has transformed the treatment of many different cancers. Immunotherapeutic agents include ipilimumab, which is a monoclonal antibody that inhibits cytotoxic T-lymphocyte associated protein 4 (CTLA-4) and nivolumab, which is a monoclonal antibody against programed death-1 (PD-1). Currently, combination immunotherapy with ipilimumab and nivolumab is widely used in various cancers, including renal cell carcinoma, metastatic melanoma and non-small cell lung cancer. However, there is currently limited evidence for the efficacy of immunotherapy in ACC, and the data is often conflicting (6–12). This case discusses the challenges of diagnosing ACC and describes a patient who had an excellent response to ipilimumab and nivolumab.

## Case description

A 32 year old woman attended the Accident and Emergency department with severe right sided abdominal pain. She had mild fatigue, but no urinary symptoms, weight loss, loss of appetite or fever. She had well-controlled type 1 diabetes, but no other significant past medical history. Her performance status was 0. She was normotensive, with no clinical features of Cushing's syndrome, hyperaldosteronism or high testosterone levels.

Her blood results were within normal limits apart from a slightly raised CRP (10mg/L, reference range: < 5 mg/L), alkaline phosphatase (138 U/L, reference range: 30 – 130 U/L) and glucose (12.9 mmol/L, reference range: 3.0 – 7.7 mmol/L). Her cortisol was normal but no other hormone profile was checked.

**Figure 1:**
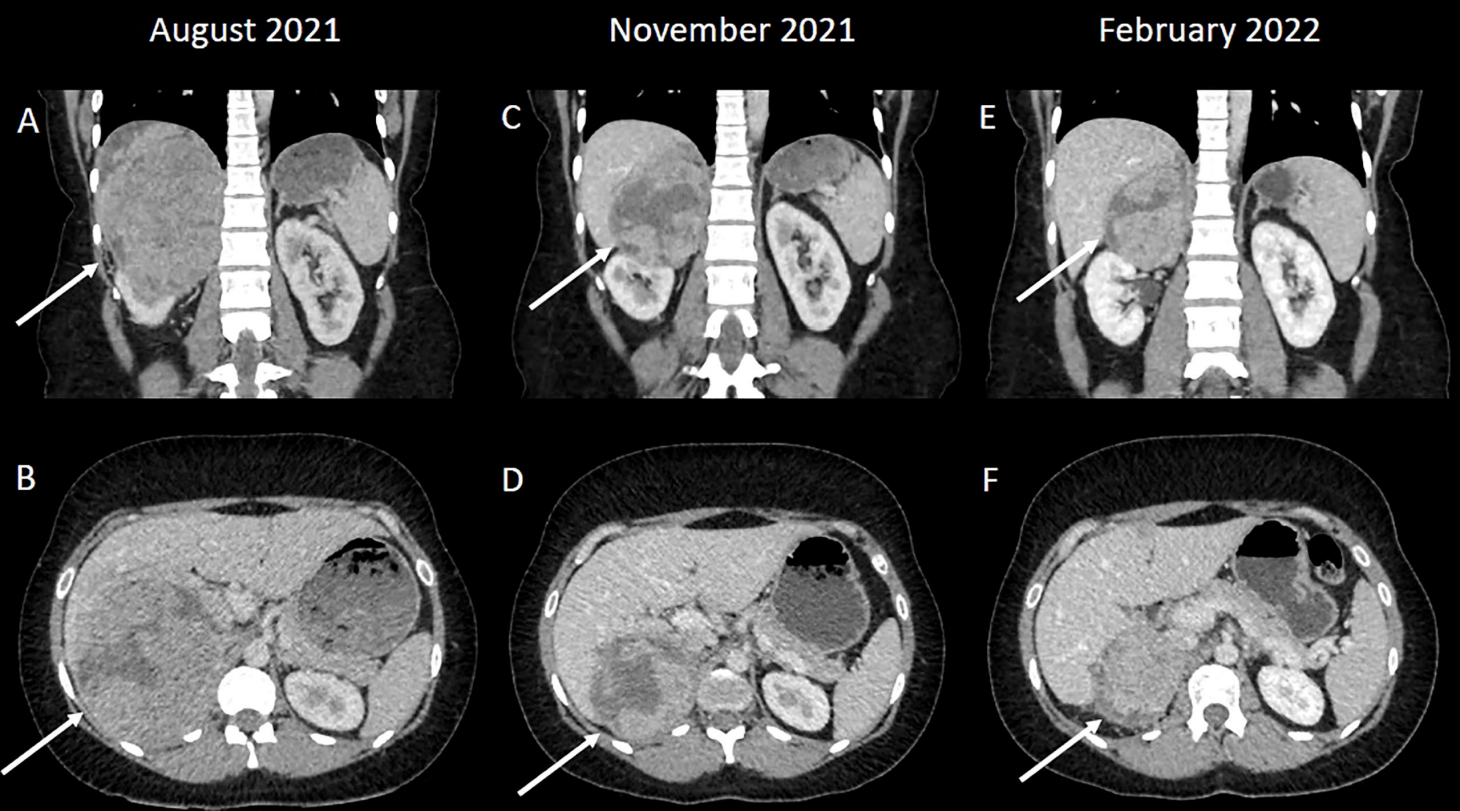
**(A, B)** A large heterogeneously enhancing mass was suspected to arise from the upper pole of the right kidney; a normal right upper renal pole could not be appreciated separate to the mass. **(C, D)** The mass has decreased in size following systemic therapy consistent with a good partial response to treatment. **(E, F)** A further reduction in size following additional systemic treatment.

**Figure 2:**
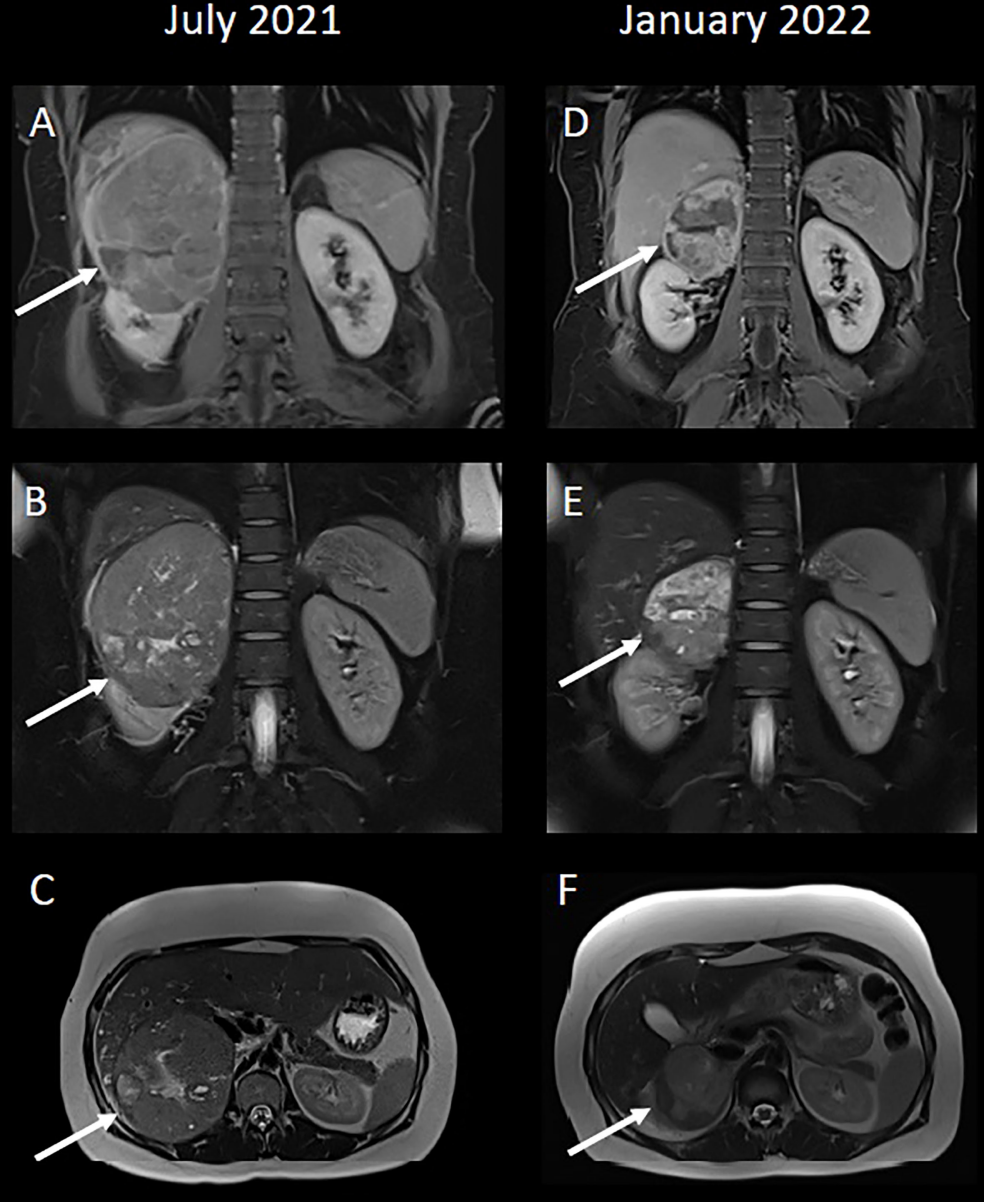
**(A, D)** Coronal T1-weighted VIBE-Dixon images, 5 minutes post-contrast. **(B, E)** Coronal T2-weighted HASTE fat-saturated images. **(C, F)** Axial T2-weighted HASTE images. **(A–C)** A large mixed signal mass, again suspected to arise from the upper pole of the right kidney. 2A demonstrates contrast-enhancement within the liver parenchyma adjacent to the mass, which was initially suspected to represent hepatic parenchymal invasion. **(D–F)** A significant reduction in size to the mass post-treatment. Following systemic therapy there is the impression of a fat plane between the right kidney and the mass itself, particularly in 2D, such that the possibility of this mass arising from the adrenal gland, rather than kidney, could have been highlighted.

An ultrasound scan showed a 14 x 11 cm heterogeneously echogenic solid-appearing mass suspected to arise from the upper to mid-pole of the right kidney. A CT scan of the chest, abdomen and pelvis in the portal venous phase showed a large heterogeneously enhancing mass, again suspected to arise from the upper pole of the right kidney, with possible invasion into the right hepatic lobe both superiorly and inferiorly (**Figures 1A, B**). The right adrenal gland and right renal vein could not be appreciated separate to this large mass. There were no distant metastases. An upper abdominal MRI scan showed a lobulated mass, again suspected to arise from the upper pole of the right kidney, with broad contact to the under surface of the liver, and with suspected liver parenchymal invasion (**Figures 2A–C**).

A core biopsy showed a malignant tumour composed of cells with clear cytoplasm and marked nuclear atypia, with large hyperchromatic, pleomorphic nuclei and high mitotic count. Immunohistochemistry showed tumour cell positivity for Vimentin and AE1/AE3. It was negative for PAX8, CD10, CK7, CK20, CDX2, GATA3, OCT3/4, S100 and TTF-1. In the context of the clinical history, this was considered by the local histopathologist to be compatible with a primary clear cell renal cell carcinoma (RCC), provisionally grade 4.

Her case was discussed at the local Urology multidisciplinary team meeting (MDT) and it was felt that the tumour would be difficult to remove surgically due to the liver involvement, and there was also concern that there might be a separate liver metastasis. The MDT recommended systemic therapy and reconsideration of surgery at a later date depending on her response. Her International Metastatic Renal Cell Carcinoma Database Consortium (IMDC) score was 1 (intermediate risk) as her time from diagnosis to systemic therapy was less than 1 year. At the time, the treatment options funded by the United Kingdom’s National Health Service (NHS) for RCC were ipilimumab plus nivolumab, axitinib plus avelumab or single agent tyrosine kinase inhibitors. Following discussions with the patient, it was decided to proceed with ipilimumab and nivolumab as it was felt that this was the treatment option that was most likely to result in sufficient tumour shrinkage to permit future surgery.

After informed consent, she commenced treatment with ipilimumab 1mg/kg and nivolumab 3mg/kg every three weeks. She developed immunotherapy-related hypothyroidism, which was treated with levothyroxine, but otherwise tolerated treatment well.

A CT scan after four cycles of ipilimumab and nivolumab showed a partial response to treatment (**Figures 1C, D**). She was re-discussed at the local Urology MDT and the surgical team felt that surgery was likely to be challenging as the renal mass was adherent to the liver. She was discussed at the regional Hepatobiliary MDT and a joint procedure involving both the urology and liver surgeons was planned. Whilst the MDT discussions and surgical work-up was ongoing, the patient completed two cycles of single agent nivolumab (480mg every 4 weeks) (**Figures 1E, F** and **2D–F**).

As part of the pre-operative process, her biopsy was reviewed by the tertiary centre. It was felt that other features of a clear cell RCC, such as a fine vascular network, were not present. Therefore, a further panel of immunohistochemistry was performed, which showed tumour cell positivity for Synaptophysin, Calretinin, Melan-A and Inhibin. EMA and Chromogranin were negative. PAX-8 was repeated which was negative. This immunophenotype was considered to be more typical of an adrenal cortical tumour, rather than a clear cell RCC (13). SF-1 was not tested as this was not available. The combined morphology and immunophenotype were favoured to represent a malignant adrenal cortical tumour (adrenal cortical carcinoma).

Routine clinical urine steroid profile showed a relative increase of androsterone and aetiocolanolone, as well as DHA metabolites. There was also an increase of cortisol (11-hydroxy) versus cortisone (11-oxo) metabolites and of 5b- versus. 5a-reduced metabolites, and a small relative increase of the 11-deoxycortisol metabolite tetrahydro-11-deoxycortisol (73ug/L) and of 16-epiandrosterone (56 ug/L). There were no accompanying increases of other specific ACC markers seen.

**Figure 3:**
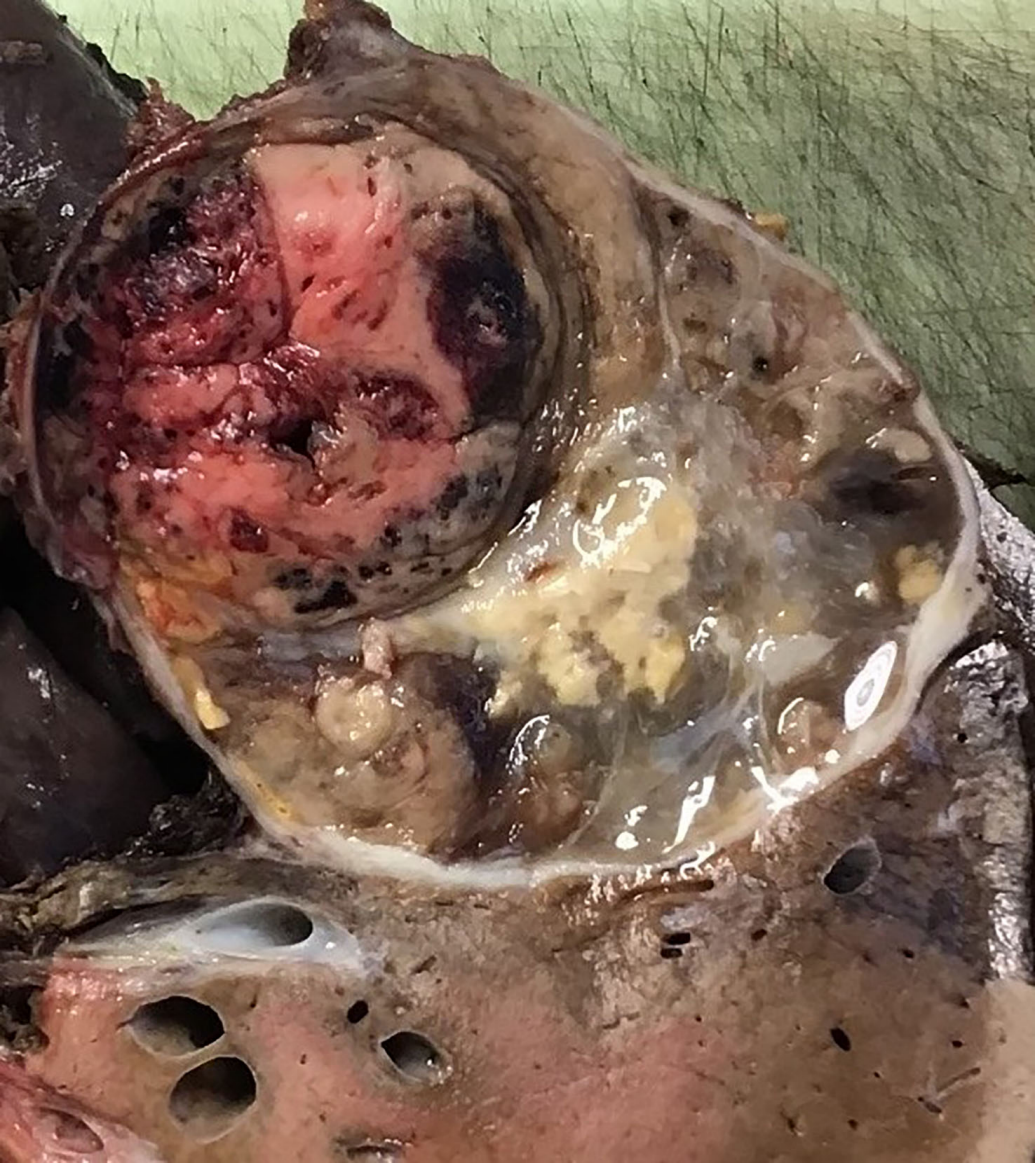
Right hepatectomy with en-bloc adrenalectomy showing an encapsulated tumour which was adherent to the liver. The tumour has a variegated appearance with solid, oedematous, haemorrhagic and necrotic areas. The tumour did not appear to invade the liver parenchyma macroscopically.

The patient had a right hepatectomy and right adrenalectomy, and resection of her right kidney was not required. Dissection around the inferior vena cava was challenging, with poor tumour planes and desmoplastic reaction but the tumour was completely excised. The excision specimen revealed an encapsulated tumour which was adherent to the liver (**Figure 3**).

**Figure 4:**
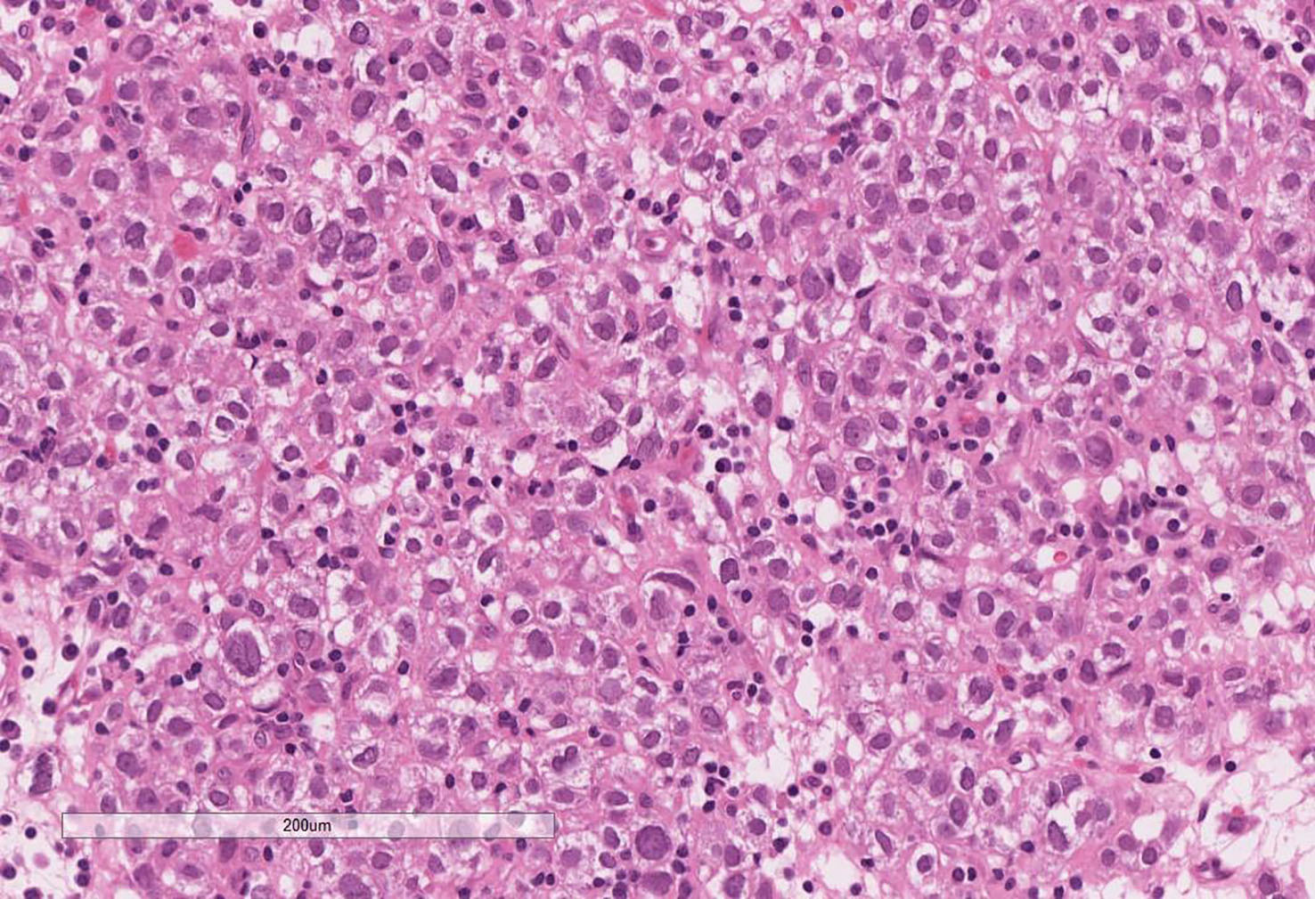
Excision of the adrenal tumour showing features consistent with an adrenal cortical carcinoma. Note the clear cytoplasm, marked nuclear atypia and patchy lymphoplasmacytic infiltrate. Areas of cells with eosinophilic cytoplasm, frequent mitoses, confluent necrosis, oedema, hyalinisation and haemorrhage were noted in other sections.

On microscopic assessment, the tumour showed features similar to those seen in the pre-operative biopsy and consistent with an ACC (**Figure 4**). The tumour showed multiple nodules of atypical cells with clear and eosinophilic cytoplasm, marked nuclear atypia and frequent mitoses. Areas of confluent necrosis, oedema, hyalinisation, haemorrhage and patchy lymphoplasmacytic infiltrate were also present. Modified Weiss score was 5 (≥3 indicates aggressive/malignant behaviour) (13). The final staging was pT3 Nx. Mismatch repair (MMR) protein immunohistochemistry showed retained staining for MLH1, PMS2, MSH2 and MSH6 within the tumour cell nuclei and therefore there was no immunohistochemical evidence of MMR deficiency.

Following discussion at the Urology MDT, the options of adjuvant mitotane, continuing nivolumab for one year or close surveillance were discussed with the patient. The patient decided to restart nivolumab, and this was restarted 10 weeks post-operatively. The patient has now completed a year of nivolumab and the most recent CT scan (12 months from surgery) shows no evidence of recurrence.

## Discussion and conclusion

This case highlights the potential pitfalls of a clear cell tumour. This tumour was initially considered to be compatible with a high-grade clear cell RCC based on morphology and immunohistochemistry. The typical immunoprofile for a clear cell RCC includes positivity for AE1/AE3, PAX-8, CD10 and Vimentin, with rare expression of CK7 (14). The biopsy showed positivity for Vimentin and AE1/AE3, which in the context of the clinical history (suspicious for a primary renal carcinoma), was considered supportive for a diagnosis of clear cell RCC.

However in hindsight, the immunoprofile, although supportive, was not diagnostic of clear cell RCC. Clear cell RCCs are virtually always positive for PAX-8 and CD10 (13), which this tumour was not. However, there is some evidence that a negative PAX-8 does not entirely exclude the possibility of a clear cell RCC in the correct context (15), which highlights the diagnostic challenge this case presents and the importance of detailed and clear clinical information for forming a differential diagnosis of a clear cell tumour.

The main differential in this case lay between a clear cell RCC and an ACC. However, other differential diagnoses to be considered in cases of ACC, include hepatocellular carcinoma and phaeochromocytoma. Adrenal cortical tumours are rare, and are therefore less likely to be considered as a differential if the clinical information, morphology and immunohistochemistry supports a renal primary.

Of note in this case, the patient received immunotherapy, which is not commonly used to treat ACC. The specific resultant effects of ipilimumab and nivolumab on the histological appearance of an ACC is not currently described in the literature. Generally, immunotherapy induces tumour changes such as necrosis and lymphoplasmacytic infiltrate, which is seen in this case. Given the possibility of some of the features being secondary to immunotherapy-induced effects, it is not clear whether a Modified Weiss score is an appropriate diagnostic tool in this setting, due to the inclusion of features such as necrosis. The Modified Weiss score helps to assign a tumour to either a ‘benign’ or ‘malignant’ category based on morphological features, and is most useful in borderline cases (13). However, in this case, as the morphological features were consistent with a malignant tumour, inclusion of post-immunotherapy features to the Modified Weiss score would not have altered the final diagnosis of an ACC.

As mentioned previously, immunotherapy is not currently a standard treatment for ACC and there is limited available evidence for its effectiveness. A subgroup analysis of the CA209-538 trial looked at combination immunotherapy with ipilimumab and nivolumab in patients with ACC (6). Six patients received treatment in the study and results showed that the objective response rate (ORR) was 33%, which is higher than the current response rate of 23% associated with mitotane-based therapy (4, 5).

The only other data for ipilimumab and nivolumab comes from a phase II trial in advanced rare genitourinary cancers including ACC (7). The ACC cohort of this trial was small (18 patients), and in contrast to the previous study, the results from this were not as promising. Only one patient had a response to treatment, giving an ORR of only 6%, although seven patients had stable disease.

The efficacy of single agent immunotherapy has also been explored in ACC. A phase II trial which treated 10 patients with ACC with single-agent nivolumab was disappointing, with a median progression-free survival of only 1.8 months, although one patient had stable disease for 48 weeks (8). Another phase II study evaluated the use of the anti-PD-1 monoclonal antibody pembrolizumab in 39 patients with advanced ACC (9). The results of this study were more encouraging, with a response rate of 23% and disease control rate of 52%. However, again this data contrasts with the results of a smaller phase II study of 16 patients treated with pembrolizumab, which showed a lower ORR of 14% (10). There is also some retrospective data suggesting a possible synergistic effect of pembrolizumab with mitotane, as in a small study of six patients, two patients had a partial response and four had stable disease (11).

The largest study of immunotherapy in ACC was the JAVELIN phase Ib trial, which treated 50 patients with previously treated ACC with avelumab (12). This demonstrated an ORR of 6% and a disease control rate of 48% (12). However, this data is difficult to interpret as 50% of patients received concomitant mitotane. In addition, patients had received a median of two previous lines of treatment (range 1-6) and better outcomes were seen in patients who had received fewer prior lines of therapy.

Therefore the data for the efficacy of immunotherapy in ACC shows variable results, with ORR ranging from 6 – 33% (see **Table 1**). Clinical trials in ACC are challenging, due to the rarity of the disease and the trial populations are generally small and heterogenous, with patients having received different amounts of previous treatment.

**Table 1:** Evidence for immunotherapy in ACC.

Trial	Phase	Immunotherapy regime	Number of patients	Response rate
Klein et al. CA209-538 (subgroup analysis) (6)	II	Ipilimumab + nivolumab	6	33%
McGregor et al. NCT03333616 (7)	II	Ipilimumab + nivolumab	17	6%
Carneiro et al. NCT02720484 (8)	II	Nivolumab	10	0%
Raj et al. NCT02673333 (9)	II	Pembrolizumab	39	23%
Habra et al. NCT02721732 (10)	II	Pembrolizumab	16	14%
Head et al. (11)	Retrospective	Pembrolizumab + mitotane	6	33%
Le Tourneau et al. JAVELIN NCT01772004 (12)	Ib	Avelumab	50	6%

There is some evidence that selected patients might benefit from immunotherapy, and so biomarkers to identify these patients would be helpful. However, again the results in ACC have been conflicting. For example, microsatellite instability is a known biomarker that can predict response to immunotherapy in other tumour types. The two patients in the CA209-538 trial who achieved an objective response both had a microsatellite unstable phenotype (6). However, our case had no evidence of microsatellite instability, and 78% of patients with ACC who responded to pembrolizumab had microsatellite stable tumours (9).

In conclusion, this case illustrates the challenges of diagnosing ACC and the importance of considering it in the initial differential diagnosis. The role of immunotherapy in ACC has been thought to be limited, however our case shows significant clinical efficacy with ipilimumab plus nivolumab in a patient with ACC. More research is needed to see if combination immunotherapy is more effective than single agent immunotherapy in this rare disease and biomarkers are also needed to improve patient selection.

## Patient perspective

When the doctor told me I had a massive tumour on my right kidney and it was invading my liver I was so shocked and upset. I started treatment in September 2021 and the only side effects that I had was a problem with my thyroid. Treatment went really well and the tumour shrunk a lot so when it came round to the operation the doctor said we might be able to keep the kidney and he would know more when he opened me up. After the operation they told me that they saved the kidney and it was on the adrenal gland. I was so happy that I’ve still got my kidney. A year has passed since my operation and I’m feeling fantastic.

## Data availability statement

The original contributions presented in the study are included in the article/supplementary material. Further inquiries can be directed to the corresponding author.

## Ethics statement

Written informed consent was obtained from the individual(s) for the publication of any potentially identifiable images or data included in this article.

## Author contributions

RC, DM and SM did the literature search and drafted the manuscript. AP reviewed the radiology images. AB reviewed the pathology. OH and NK performed the patient’s surgery. SM treated the patient with ipilimumab and nivolumab. All authors read and approved the final manuscript.

## References

[1] Gatta G Capocaccia R Botta L Mallone S De Angelis R Ardanaz E. Burden and centralised treatment in Europe of rare tumours: results of RARECAREnet - a population-based study. Lancet Oncol (2017) 18(8):1022–7. doi: 10.1016/S1470-2045(17)30445-X 28687376

[2] Fassnacht M Assie G Baudin E Eisenhofer G de la Fouchardiere C Haak HR. Adrenocortical carcinomas and Malignant phaeochromocytomas: ESMO-EURACAN Clinical Practice Guidelines for diagnosis, treatment and follow-up. Ann Oncol (2020) 31(11):1476–90. doi: 10.1016/j.annonc.2020.08.2099 32861807

[3] Johanssen S Hahner S Saeger W Quinkler M Beuschlein F Dralle H. Deficits in the management of patients with adrenocortical carcinoma in Germany. Dtsch Arztebl Int (2010) 107(50):885–91. doi: 10.3238/arztebl.2010.0885 PMC3021904 21246024

[4] Megerle F Herrmann W Schloetelburg W Ronchi CL Pulzer A Quinkler M. Mitotane monotherapy in patients with advanced adrenocortical carcinoma. J Clin Endocrinol Metab (2018) 103(4):1686–95. doi: 10.1210/jc.2017-02591 29452402

[5] Fassnacht M Terzolo M Allolio B Baudin E Haak H Berruti A. Combination chemotherapy in advanced adrenocortical carcinoma. N Engl J Med (2012) 366(23):2189–97. doi: 10.1056/NEJMoa1200966 22551107

[6] Klein O Senko C Carlino M Markman B Jackett L Gao B. Combination immunotherapy with ipilimumab and nivolumab in patients with advanced adrenocortical carcinoma: a subgroup analysis of CA209-538. OncoImmunology (2021) 10(1):1908771. doi: 10.1080/2162402x.2021.1908771 33889439 PMC8043165

[7] McGregor B Campbell M Xie W Farah S Bilen M Schmidt A. Results of a multicenter, phase 2 study of nivolumab and ipilimumab for patients with advanced rare genitourinary Malignancies. Cancer. (2020) 127(6):840–9. doi: 10.1002/cncr.33328 PMC13213840 33216356

[8] Carneiro B Konda B Costa R Costa R Sagar V Gursel D. Nivolumab in metastatic adrenocortical carcinoma: results of a phase 2 trial. J Clin Endocrinol Metab (2019) 104(12):6193–200. doi: 10.1210/jc.2019-00600 31276163

[9] Raj N Zheng Y Kelly V Katz S Chou J Do R. PD-1 blockade in advanced adrenocortical carcinoma. J Clin Oncol (2020) 38(1):71–80. doi: 10.1200/jco.19.01586 31644329 PMC7351334

[10] Habra M Stephen B Campbell M Hess K Tapia C Xu M. Phase II clinical trial of pembrolizumab efficacy and safety in advanced adrenocortical carcinoma. J ImmunoTherapy Cancer (2019) 7(1):253. doi: 10.1186/s40425-019-0722-x PMC6751592 31533818

[11] Head L Kiseljak-Vassiliades K Clark TJ Somerset H King J Raeburn C. Response to immunotherapy in combination with mitotane in patients with metastatic adrenocortical cancer. J Endocr Soc (2019) 3(12):2295–304. doi: 10.1210/js.2019-00305 PMC6853671 31745526

[12] Le Tourneau C Hoimes C Zarwan C Wong DJ Bauer S Claus R. Avelumab in patients with previously treated metastatic adrenocortical carcinoma: phase 1b results from the JAVELIN solid tumor trial. J ImmunoTherapy Cancer (2018) 6:111. doi: 10.1186/s40425-018-0424-9 PMC6198369 30348224

[13] Lloyd RV Osamura RY Klöppel G̈nter Rosai J Bosman FT Jaffe ES. WHO classification of tumours of endocrine organs. 4th Edition Vol. 10. . Lyon: International Agency for Research on Cancer (2017).

[14] Moch H Humphrey PA Ulbright TM Reuter VE eds. WHO classification of tumours of the urinary system and male genital organs. 4th Edition. Lyon, France: International Agency for Research on Cancer (2016).

[15] Barr ML Jilaveanu LB Camp RL AdenIran AJ Kluger HM Shuch B. PAX-8 expression in renal tumours and distant sites: A useful marker of primary and metastatic renal cell carcinoma? J Clin Pathol (2014) 68(1):12–7. doi: 10.1136/jclinpath-2014-202259 PMC4429054 25315900

